# A case study of interdisciplinary thematic learning curriculum to cultivate “4C skills”

**DOI:** 10.3389/fpsyg.2023.1080811

**Published:** 2023-03-07

**Authors:** Peiqi Ye, Xionghu Xu

**Affiliations:** ^1^Xiamen Xingnan Middle School, Fujian, China; ^2^School of Fine Arts, East China Normal University, Shanghai, China; ^3^School of Communication and Electronic Engineering, East China Normal University, Shanghai, China

**Keywords:** interdisciplinary thematic learning, critical thinking, communication, creativity, collaboration

## Abstract

Critical thinking, communication, collaboration, and creativity are four fundamental skills for students in the 21st century, indicating the way for nurturing talents required for future social development. Interdisciplinary thematic learning has become an important educational carrier for “4C Skills” training, with its connotation coinciding with the training requirements of “4C Skills.” Few academics, however, have looked into interdisciplinary thematic learning activities based on real-world problems. In this study, using a middle school in Xiamen, Fujian Province as an example, 32 s-year students in middle school were given several problem-solving tasks relevant to “visual disaster weather.” Based on test coding and questionnaire evaluation, class notes, course videos, student solutions, and interview texts, we examined the development of students’ 4C skills through real-world problem-based interdisciplinary thematic learning activities. This study discovered that an interdisciplinary thematic learning environment centered on real-world challenges fosters students’ creative thinking in open practice while also encouraging group communication and collaboration. Students also gain critical thinking skills through questioning and critique.

## 1. Introduction

With the continual advancement of information technology, the rapid entrance of Industry 4.0, and globalization, the globe is developing a close community of interests, and humanity is confronted with new difficulties. Real-world problems are frequently multidisciplinary and arise in complicated systems (Dym et al., 2005). These issues are frequently intertwined with systems and necessitate complicated problem-solving talents, ingenuity, and the participation of various parties (Levy, 1992; Richardson et al., 2001). The focus of education has switched from gaining superficial knowledge to developing a wide range of essential competencies. As a result, many academics are concerned about how to properly cultivate students’ key competencies for the future age.

The project “The Definition and Selection of Competences: Theoretical and Conceptual Foundations (DeSeCo)” of Organization for Economic Cooperation and Development (n.d.) was launched in late 1997.The OECD defines core competency as “the ability to mobilize psychological and social resources in a specific setting, including skills and attitude to deal with complicated problems.” The three-level frame structure “core accomplishment - ability index - behavior description” as shown in Table 1.

**Table 1:** The three-level frame structure “core accomplishment–ability index–behavior description”.

Literacy classification	Key literacy
Use tools interactively	Use language, symbols, and text interactively
	Use knowledge and information interactively
	Use technology interactively
Interact in heterogeneous groups	Build good relationships with others
	teamwork
	Managing and resolving conflicts
Act autonomously	Act in a complex environment
	Form and execute a personal plan or life plan
	Protect and defend rights, interests, restrictions and needs

These categories, each with a specific focus, are interrelated, and collectively form a basis for identifying and mapping key competencies. The need for individuals to think and act reflectively is central to this framework of competencies. Reflectiveness involves not just the ability to apply routinely a formula or method for confronting a situation, but also the ability to deal with change, learn from experience and think and act with a critical stance.

The EU updated the core literacy framework in 2018, and it now includes eight components: “literary literacy,” “multilingual literacy,” “mathematical literacy,” “science and technology literacy,” “engineering literacy,” “digital literacy,” “personal, social and learning literacy,” “citizenship literacy,” “innovation and entrepreneurship literacy,” and “cultural awareness and expression.” The EU’s basic literacy framework is more detailed than the OECD’s. Directions for knowledge, skill, and attitude are clearer, and both disciplines and cross-disciplines are considered. It may have explicit discipline attribution or pervade the learning of several fields (European Council, 2018).

**Figure 1:**
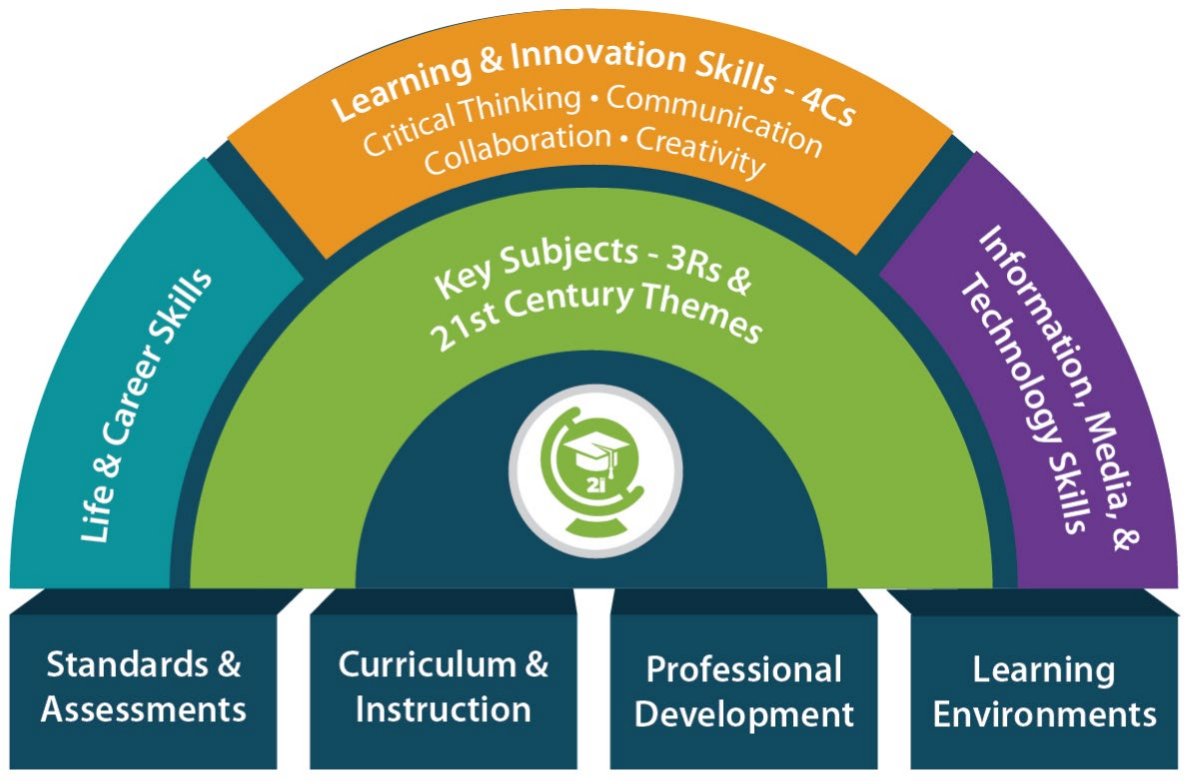
p21 Core literacy framework.

As shown in Figure 1, Partnership for 21st Century Skills Learning (P21) proposed the “4C Skills” (critical thinking, communication, cooperation, and creativity) in 2007 (Partnership for 21st Century Skills, 2007). While distinguishing between interdisciplinary themes and core topic learning, the “4C Skills” will also organically interconnect and mutually support subject material knowledge and core skills. The 4C abilities can help students not only achieve in all areas of formal education, but also adapt to an ever-changing world (Partnership for 21st Century Learning, 2015).

Various countries have begun to debate ways to help students understand the “island of disciplines” “fractured disciplinary knowledge through interdisciplinary learning and curriculum integration. Interdisciplinary learning can break through academic borders to enhance students’ fundamental competencies by merging course design content with cross-border teaching and implementation (Xinning and Xinyang, 2013). It can also combine students’ learning in order to connect their knowledge structure, forming structured knowledge, and thinking in order to fully comprehend the objective world and solve practical difficulties (Meng, 2022). Despite the fact that interdisciplinary learning has received a lot of attention and is regarded as an efficient technique to develop fundamental competences, it still has a lot of obstacles. The major difficulty is determining how to improve the systematic and structured nature of the interdisciplinary learning curriculum. Furthermore, given the dominance of traditional curriculum in the educational system, there are numerous questions concerning how to evaluate the success of integrated teaching and students’ interdisciplinary abilities (Shen et al., 2014; Griese et al., 2015; Herro et al., 2017; You et al., 2018). Researchers discovered numerous barriers to the implementation of interdisciplinary learning in schools in terms of pedagogy, curriculum, and structure. It is indicated that there is an urgent need to provide pedagogical aid and curriculum resources to enable teachers to utilize Interdisciplinary Learning.

Therefore, this study was driven by the following research questions:

1. Do students promote the development of the 4C skills in interdisciplinary topic learning?
2. How do these competencies develop across the interdisciplinary thematic learning?

We thoroughly interpreted the connotation of interdisciplinary learning and 4C skills, designed interdisciplinary thematic learning activities, and introduced interdisciplinary thinking to break the boundary and realize deep multidisciplinary integration using interdisciplinary themed activities as the core content. Through interdisciplinary thematic learning activities, this study attempted to enable participants to improve 4C abilities and integrate thinking. This paper identifies the role of activities in interdisciplinary thematic learning courses and investigates the elements of activities based on the learning situation and participant performance, as well as activity theory, in order to gain a better understanding of the construction and evaluation mechanism of an interdisciplinary thematic learning curriculum system. We strive to provide novel concepts and approaches for curriculum design, development, and assessment of interdisciplinary subject learning, which has major theoretical and practical consequences for talent development in the current era.

## 2. Literature review

### 2.1. Based on real-world problems

The challenges we face in the real world are complex, and solving them requires the integration of many disciplines, concepts, and talents (Weng et al., 2022). The interdisciplinary nature of real-world problem-based learning, as opposed to the disciplinary framework of formal education, underpins arguments for curriculum integration (Jacobs, 1989; Beane, 1995; Czerniak et al., 1999). As a result, learning through real-world challenges is a good way for students to overcome complex problems (Ferreira and Trudel, 2012). More emphasis is placed on learners’ ability to apply knowledge to make decisions and solve issues, as well as the capacity to evaluate when and how to obtain more information, than on memorizing material or procedures (Pellegrino and Hilton, 2012). Furthermore, learning exercises focused on real-world challenges boost students’ motivation and inspire them to experiment over time. Students may only strengthen their creative and critical thinking abilities by being exposed to unsolvable open-ended problems and real-world disciplinary experience (Helle et al., 2006).

It has been discovered that learning exercises based on real-world challenges motivate students and improve their ability to learn tough subject as well as problem-solving skills and confidence (Kokotsaki et al., 2016). Relevant research has shown that learning approaches based on real-world challenges can boost students’ creative and critical thinking skills (Chan, 2013). The problem-based learning technique was used for one semester with higher education visual arts students to see how it improved their creative and critical thinking dispositions. Finally, studies show that problem-based learning has a significant impact on creative thinking (Ulger, 2018). Another study will look into the attitudes, inventiveness, and critical thinking of students who have been exposed to problem-based learning, as well as their relevance to nursing education and clinical practice (Pardamean, 2012). Based on the literature review, we found that learning activities based on real-world problems can improve students’ 4C skills. However, previous studies mainly focus on a single discipline without investigating interdisciplinary learning activities. In the present study, whether interdisciplinary learning activities based on real-world problems can promote the development of students’ 4C skills was explored.

### 2.2. Activity theory

The study of how humans learn is known as learning theory. We can only improve teaching if we understand the learning process better. In the course of developing learning theory, the core premise of “learning is a stimulus–response” is gradually abandoned, and more and more emphasis is devoted to learner self-construction and interaction with the learning environment and learning community. The activity hypothesis goes into greater detail about the process and organization of this relationship Igira and Gregory (2009). Teaching practice has changed in tandem with learning theory, from classic lecturing-based teaching to student-centered teaching to the present emphasis on designing teaching and learning activities to improve students’ 4C skills Yushun et al. (2022). As a result, activity theory offers a valuable perspective for studying teaching activities and assessing classroom performance (Engeström, 1987).

**Figure 2:**
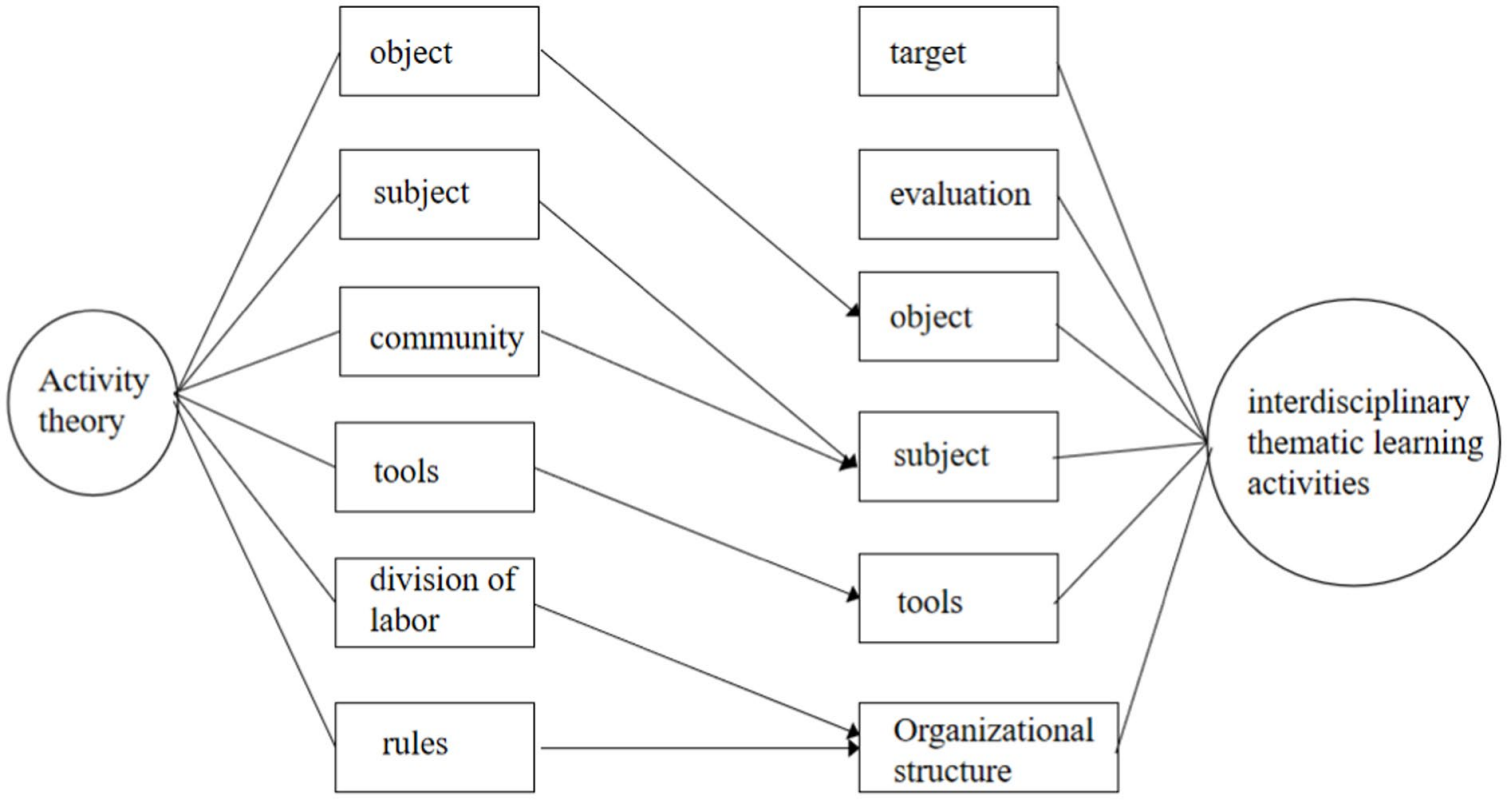
Six elements of interdisciplinary thematic learning activities.

According to Nardi (1996), AT is an effective method for studying professional behavior because it adheres to an object-focused mentality in the sense of a goal, a motive, or a result, allowing coherence and concentration to guide decisions and actions. Furthermore, AT maintains close linkages with practice to ensure that relevant research has an impact. According to activity theory, the smooth execution of activities necessitates the division of labor between the subject and the society, as well as the laws of its operation (Tessier and Zahedi, 2022). The organizational forms of teaching and learning activities, including competition, cooperation, practice, and other forms, fully reflect the norms of activities and the division of labor between subjects in the field of teaching and learning (Sannino and Ellis, 2014). To summarize, Figure 2 depicts the relevant relationship between the elements of interdisciplinary thematic learning activities in this study and the elements of Activity theory.

Interdisciplinary topic learning necessitates a large number of learning activities or tasks. As a result, the comprehensive study of interdisciplinary subject learning based on activity theory provides a novel theoretical research and practical approach for assessing interdisciplinary topic learning.

### 2.3. Interdisciplinary thematic learning

Different phrases (for example, integrated practice and interdisciplinary) are used in education to describe the connection and integration of multiple disciplines (Roehrig et al., 2021). Professor Woodworth of Columbia University coined the word “interdisciplinary” in 1926. It refers to practical activities involving two or more disciplines that extend beyond the boundaries of a given discipline in order to allow learners to unite their knowledge and skills. Interdisciplinary learning combines two or more disciplines, ignoring the nature of each discipline and focusing instead on the links between them.

Interdisciplinary thematic learning, unlike interdisciplinary learning, can be broadly infused into all courses as a curriculum concept and teaching/learning method or as a special curriculum form an independent interdisciplinary curriculum that transcends the logical system of the discipline itself (Zhanghua, 2017). Interdisciplinary thematic learning focuses on obtaining knowledge and skills in interdisciplinary thematic content by emphasizing the correlation between subjects across disciplines. Interdisciplinary thematic learning frequently connects social, political, economic, international, and environmental issues through a topic or real-world situation Wang et al. (2020). Interdisciplinary thematic learning is a process of constructing comprehensive learning activities, focused on a specific research topic and discipline curriculum content, and combining relevant knowledge and methodologies from other courses, based on students’ learning basis (Gao et al., 2020). During the theme inquiry, this approach allows students to think across borders, exposes them to interdisciplinary knowledge integration and building, and strengthens their problem-solving abilities. Interdisciplinary thematic learning highlights important topics, questions, or issues that need to be solved in the real world and extends every subject for structured and meaningful learning, allowing students to spend enough time exploring, thinking deeply, and constructing knowledge content systems Nakakoji and Wilson (2020). Students combine knowledge and abilities from numerous disciplines during the learning process, delve deeply into topics, and participate in varied activities, promoting core competencies (Gardner et al., 2003). Although problem-based interdisciplinary learning seems promising in promoting student cognitive development, research and educational practices that apply problem-based interdisciplinary teaching are insufficient, particularly qualitative research on the development of students’ 4C skills (Papadakis et al., 2022).

### 2.4. 4C skills

Although many experts disagree on the precise definition of skills in the 21st-century (Voogt and Roblin, 2012; Van Laar et al., 2017), 4C skills have been identified as critical competences (Silber-Varod et al., 2019). All four talents are related to cognitive capacities of learners, although with distinct emphasizes. Critical thinking, for example, is a cognitive strategy critical to improving students’ decision-making, critical judgment, and self-reflection abilities (Pintrich et al., 1991); it is a thinking ability that extends beyond the boundaries of disciplines and requires learners to think in new ways and connect different disciplines (Hu et al., 2020). Creativity is a process in which students produce their own ideas or answers (Yang and Cheng, 2010); it is a highly comprehensive and encompassing core skill that intensively embraces talent. Good communication assists students to deal with challenges more effectively (Ghefaili, 2003), allowing them to overcome academic boundaries and fully comprehend the knowledge. Collaboration allows students to fully utilize collective wisdom while also combining individual abilities, expertise, and wisdom to attain common goals (Lin et al., 2020).

Relevant studies have shown that interdisciplinary thematic learning activities can boost the development of students’ 4C skills. For example, in one study, researchers prepared a two-day workshop for 15 middle school students who were new to programming (Charlton and Avramides, 2016). The Internet of Things has been utilized to support smart city initiatives in which students must address physical computing difficulties while building electrical artifacts. Researchers discovered that by participating in these learning activities, students practiced all 4C skills (English et al., 2017). Students created earthquake-resistant structures out of common materials. They planned and erected a two-story structure to survive a simulated “earthquake” in small groups. The researchers examined how students participated in the design process and utilized interdisciplinary knowledge using open coding. Students’ evaluation indicated that the assignment increased their 4C skills, such as communication and cooperative ability. A two-year interdisciplinary course was described in another study. Students spent the first year mostly on coding courses, while the second year was devoted to renewable energy. Researchers evaluated students’ creativity by collecting written materials such notes as data. According to statistical research, the multidisciplinary program boosted students’ cognition, problem-solving skills, and creativity (Chen and Lin, 2019).

Interdisciplinary thematic learning, according to the literature, serves a unique function in developing students’ intelligence, communication, and collaboration, as well as cultivating creativity. Students can integrate knowledge of society, ecology, and other disciplines through interdisciplinary thematic learning to develop their inventive consciousness, creative desire, problem-solving ability, art literacy, and moral cultivation, promoting their innovative thinking (Miller and Krajcik, 2019). Furthermore, the ability to transcend the knowledge boundaries of a certain subject has been tested through qualitative analysis in the preceding literature. Despite the use of various data for qualitative analysis, the current study failed to conduct continuous tracking and data statistics to demonstrate participants’ development in interdisciplinary thematic learning ability and to analyze statistical data on participants before and after the learning process. As a result, it is impossible to say if the learners’ improved interdisciplinary thematic ability is due to the learning program.

Based on prior research, this study used MLA (Microgenetic Learning Analysis) (Ackermann and Edith, 2013), and AT (Activity theory) to trace the trajectory of participants’ cognitive changes during learning activities (Siegler, 2007), with an emphasis on the cognitive sequence that participants use to grasp a concept. To further explore the broad association between interdisciplinary thematic learning and 4C skills, qualitative research was undertaken by collecting and evaluating data such as notes, study sheets, and interview transcripts throughout participants’ learning (Wenzheng and Qiuxuan, 2021). Furthermore, participants and researchers employed assessment measures to analyze learners’ learning before and after the course, as well as their overall learning aptitude. The MLA enables researchers to analyze not only what students know (what they know), but also the processes/patterns that occur when changes occur (how they get there), allowing them to go beyond the pre-test/post-test approach to learning.

In short, preliminary research indicates that little qualitative research or evaluation of problem-based interdisciplinary learning and the learning process in interdisciplinary thematic learning activities exists. More research is required to determine how interdisciplinary thematic learning enhances students’ competency and 4C skills Zhao and Wang (2022). As a result, a case study using qualitative data sources was used to answer research questions about the development of students’ 4C skills, such as “How do 4C skills assist students?” and “How are 4C skills developed in students’ interdisciplinary thematic learning?”

## 3. Methodology

### 3.1. Research design and context

A case study was conducted to assess whether interdisciplinary thematic learning activities improve students’ 4C skills development. The study enlisted the help of 32 students from various classes at the same high school. The study was conducted at a middle school in Xiamen, Fujian Province, China. We chose a group of students who were all at the same learning level and did not receive any after-class tutoring. A total of 32 students from each Grade 2 class were selected to participate in this interdisciplinary thematic learning assignment. There were 20 boys and 12 girls, with the majority of them being 13 years old. Before the study, we acquired vital background information from the participants, as shown in Table 2 below. Furthermore, prior to participating in research activities, selected students were asked to monitor their own learning or cooperation and were introduced to the essential concepts of interdisciplinary thematic learning and activity theory.

**Table 2:** Participant demographics.

	**At the age of 13**	**At the age of 14**	**Sum**
Male	12	8	20
Female	9	3	12
Sum	21	11	32

This case study is part of an interdisciplinary thematic learning course called “Visible Disaster Weather, “in which students are encouraged to build problem-solving and 4C skills while participating in a series of activities based on real-world challenges.

For 5 weeks, a one-hour class was held during after-school service hours on Fridays. The interdisciplinary thematic courses cover topics such as geography, biology, information technology, art, and so on. We investigated the impact of disasters on environmental sustainability and the fundamental causes of environmental problems using real-world challenges to raise learners’ awareness of social responsibility, build the notion of harmony between humans and nature, and promote learners’ 4C skills. Specifically, during the first class, participants used virtual technology to experience several types of catastrophe weather and thought critically about the negative impact of disaster weather on society. The following three classes focus on problem-solving and how to visually evaluate data, as well as how to translate disaster weather data into 3D models using visual art to communicate the ramifications of climate change and appeal to the public to protect the environment. Students were tasked to present their projects in the previous lesson. The content structure of interdisciplinary thematic learning is shown in Table 3.

**Table 3:** Participant demographics.

**Curriculum**	**Subject**	**Content**	**Driving questions and tasks**	**The ability to develop**	**Inherent problem**
Lesson 1	Geography	To understand the occurrence, development and distribution of all kinds of disaster weather in the geographical environment	What are the environmental effects of disaster weather?	Critical thinking, communication, and collaboration	How to promote sustainable development of the environment?
Lesson 2	Biology	The ecosystem causes disaster weather	How to protect ecosystems to reduce disaster weather?	Critical thinking, communication, and collaboration	
Lesson 3	Information Technology	Visual analysis of disaster weather data; 3D modeling	How to use information technology to show disaster weather?	Critical thinking, communication, collaboration, and creativity	
Lesson 4	Art	Creative practice of modeling of disaster weather	How to raise awareness of environmental sustainability through visual art expression?	Critical thinking, communication, collaboration, and creativity	
Lesson 5	Synthesis	Project presentation	How to show the innovative points of the designed 3D model?	Communication and collaboration	

**Figure 3:**
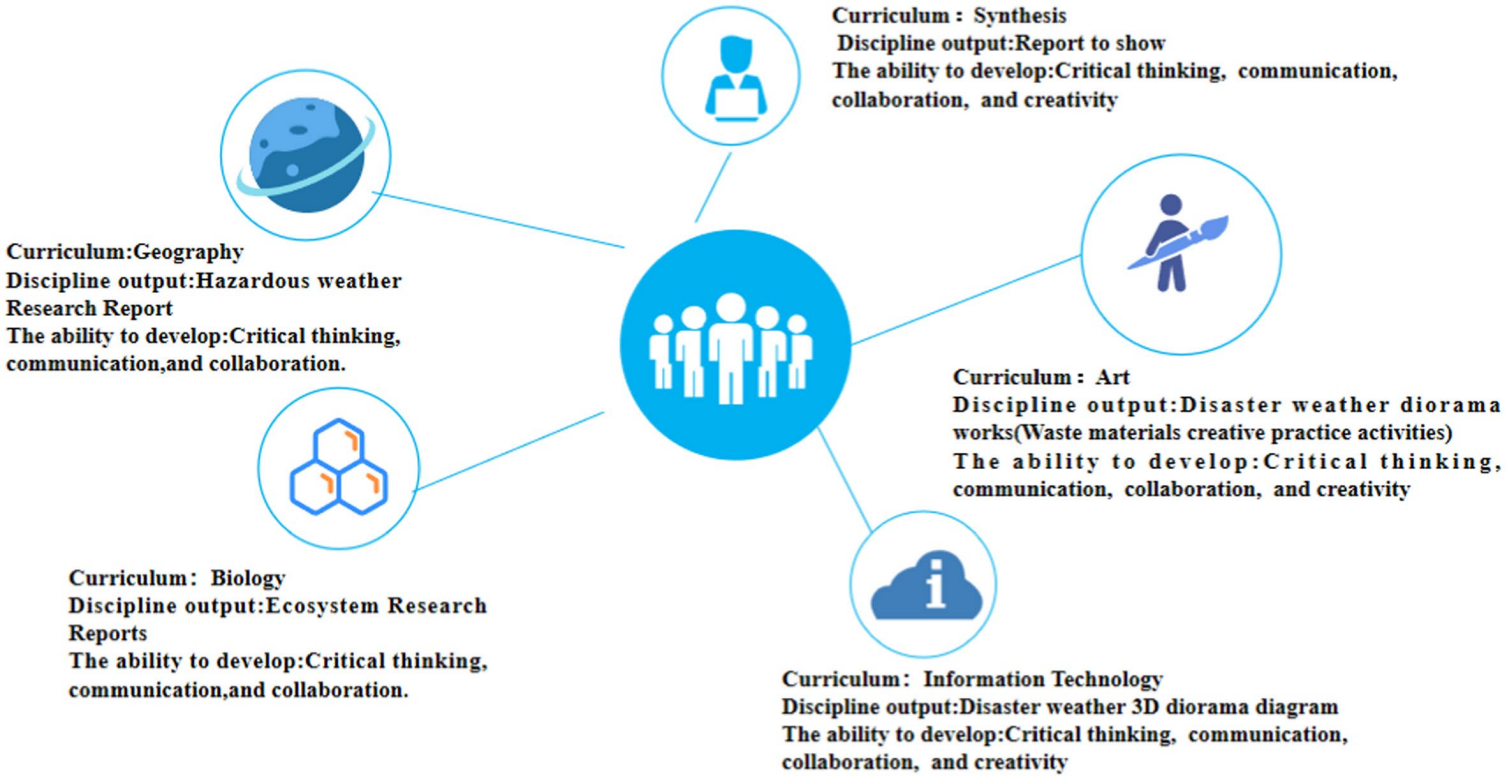
A framework for designing interdisciplinary thematic learning activities oriented to 4C skills.

Each lesson consists of whole-class teaching by the teacher and group problem-solving by the students. Subject teachers lead students through assigned questions and assignments and teach fundamental knowledge ideas. Students are given adequate time during group discussions to use the concepts and approaches they have learnt to solve challenges. Furthermore, kids are encouraged to actively collaborate with others in order to improve their communication abilities. The output outcomes of each discipline and the developed 4C skills are shown in Figure 3.

**Figure 4:**
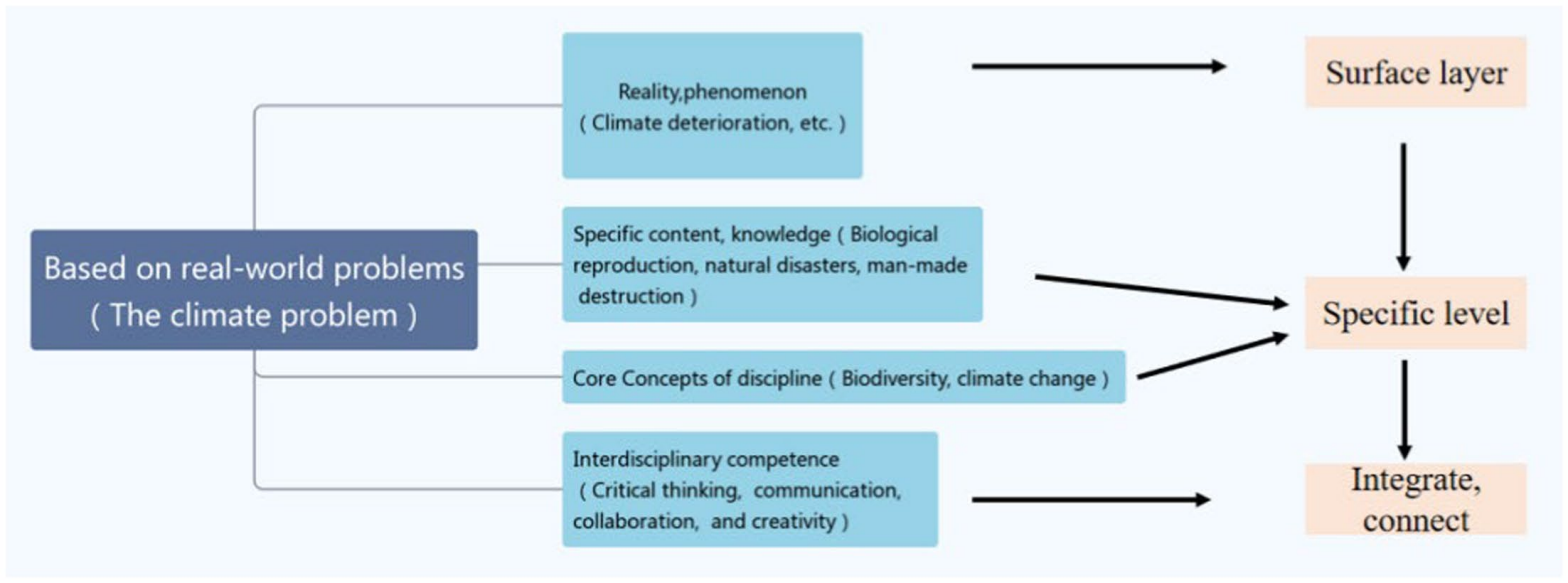
Cognitive development of interdisciplinary thematic learning.

As illustrated in Figure 4, this interdisciplinary thematic learning activity is based on real-world challenges. Taking real-world problems as the focus of an interdisciplinary study can make isolated facts or abilities meaningful and is a vital link in establishing an individual cognitive structure in a system; it can assist participants form a complex cognitive structure linked with tangible and abstract. Deep and transportable subject core notions and core competence can be abstracted on this basis. Then, questions are handled fully to establish new knowledge linkages, form new cognition and understanding, and deepen thinking. Interdisciplinary thematic learning, in a nutshell, is a dynamic notion that entails not only the appropriate integration of various knowledge elements in individual cognitive structures, but also a constant process of reflective actions to value addition and efficient usage. Real-world problems/topics serve as conceptual anchors that connect information from many disciplines. The initially separate and somewhat fragmented interdisciplinary knowledge is interconnected by merging the information and methods of two or more disciplines through themes. Themes seek common elements in interdisciplinary knowledge and integrate knowledge elements into an organic whole through connection, articulation, and reorganization, which can break down disciplinary boundaries and make teaching more focused and coherent to form a more comprehensive understanding of the subject knowledge picture.

As seen in Figure 4, the evolution follows the “truth-specific concept-subject core concepts-interdisciplinary concept” path, from surface thinking to concrete item cognition, to achieve integration of knowledge, contacts, and creation. Interdisciplinary thematic learning seeks to condense important abilities and higher-order thinking of interdisciplinary information in order to achieve core literacy.

As a “bridge, “interdisciplinary thematic learning based on the reality of issues/topics mediates and is bidirectional. First, the theme transforms students’ individual practical experience into the cognitive structure of the learning community, and then the learning community’s internal cognition is transformed outward into problem-solving capabilities with strong mobility and 4C skills. Second, knowledge from several disciplines can be linked through the theme, which serves as a “bridge” to assist students in crossing subject borders and integrating subject knowledge. Furthermore, participants in interdisciplinary topic learning have three abilities: vertical thinking, horizontal thinking, and systematic thinking. Vertical thinking entails participants being able to clarify the depth and growth of subject notions. Lateral thinking shows the cognitive functioning of interdisciplinary themed learning participants. The ability to integrate the depth of subject knowledge and the breadth of interdisciplinary knowledge in interdisciplinary thematic learning activities to form a new knowledge framework in a network manner that can be used to generate innovative solutions to teaching problems is referred to as system thinking. It can be seen that interdisciplinary thematic learning, as the “connecting hub” of interdisciplinary learning, assists participants in vertically determining the development context of the discipline’s internal knowledge system and horizontally connecting interdisciplinary concepts to form a knowledge network. Furthermore, interdisciplinary thematic learning significantly alters the typical topic and knowledge structure thinking mode. It places the cultivation of core competency first, which is followed by the course reformation “from knowledge to accomplishment.”

**Figure 5:**
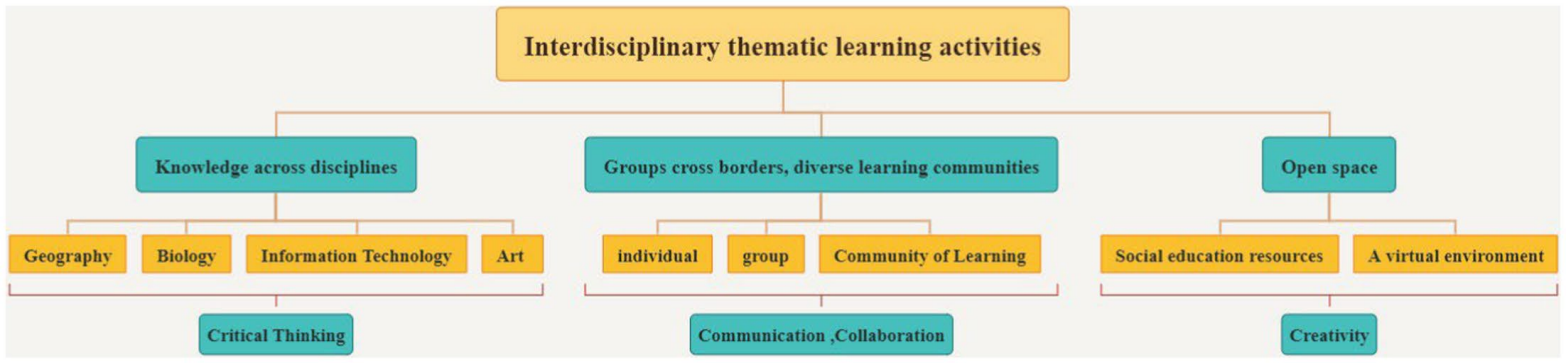
A framework for designing interdisciplinary thematic learning activities oriented to 4C skills.

This case design adheres to the curriculum design structure outlined above (Figure 5). Thematic courses emphasize the development of critical thinking skills through the use of interdisciplinary knowledge as the primary material. Thematic courses are aimed to give students more access to interdisciplinary information in theme-related domains by picking real-world challenges based on deep integration of interdisciplinary knowledge. Along with gaining interdisciplinary knowledge, the critical thinking approach of “identifying issues, creatively hypothesizing, verifying by practice, and looking for answers” is emphasized, helping students to internalize and individualize the stuff they have acquired (Zhao Huiqin and Zhaoxue, 2019). Furthermore, transboundary collaborative learning activities based on a problem shared by different individuals, groups, or project collaboration in the form of groups can create a diverse learning community, promote knowledge transfer among individuals and groups, and foster the formation of collective wisdom across groups. Students can improve their capacity to acquire information, communicate, collaborate, and solve problems by employing comprehensive knowledge in this manner. Finally, to break through traditional classrooms, flexible and diverse open spaces are built through space crossover, including museums, art galleries, and other social educational resources, as well as virtual environments across time and space built using virtual reality and augmented reality technology. Moreover, the 3D depiction of plane knowledge material through visual arts and other forms might encourage participants’ systematic thinking and deepen their cognition.

### 3.2. Data collection, selection, and analysis

We gathered relevant data from a variety of sources to investigate the process of participants’ learning experiences while learning and show the development process as well as the current condition of their 4C skills. To begin, pre-and post-tests were administered, and 5-point Likert scales (evaluation metrics) were handed to 32 students for statistical analysis in order to determine the amount of growth of students’ 4C skills. Students were asked to fill in the scales to assess their fundamental understanding of their 4C skills. Their self-reports were statistically and analytically examined. Lower ratings indicate a less positive attitude toward the development of 4C skills through interdisciplinary thematic learning, whereas higher levels indicate a more positive attitude. In addition, participants’ real learning activities and problem-solving procedures were videotaped. Text answers from students throughout interdisciplinary topic learning activities were collected, as were notes of interaction between students and their peers. After-class interviews were held. Finally, the ground theory was used to the coding analysis of text data from participants in order to determine the growth of their 4C skills.

#### 3.2.1. Pre-and post-test data collection and analysis

To determine if the level of participants’ 4C skills improved as a result of interdisciplinary thematic learning, a survey and data analysis on their 4C skills before and after interdisciplinary thematic learning were undertaken. The development standard of 4C talents is separated into five tiers, as illustrated in Table 4. Level A denotes the highest level of ability, Level B represents good ability, and Level C denotes average competence. Level D behavior characteristics are those that have not yet been acquired and must be strengthened. Level E represents behavioral features that have not been acquired and will require significant effort to strengthen.

Table 5 demonstrates that *p* = 0.00 < 0.05 in the t-test of interdisciplinary topic learning. There are considerable variations between students in critical thinking, communication, collaboration, and creativity pre-and post-tests. Moreover, the average values of the four dimensions improve following the test, indicating that students mastered specific 4C skills during the course. Furthermore, the disparities in communication and cooperation are enormous, indicating that interdisciplinary topic learning based on real-world situations can considerably improve participants’ communication and collaboration skills.

**Table 4:** Example of evaluation metrics for 4C skills through interdisciplinary thematic learning.

**Assessment criteria**	**Level**				
	**Canonical (A/4)**	**Accomplish (B/3)**	**Developing (C/2)**	**Still need to work hard (D/1)**	**It takes a lot of effort (E/0)**
Critical thinking					
Communication					
Collaboration					
Creativity					

There were 32 valid assessment scales collected in total. The results of an independent sample *t*-test on the four evaluation indicators are reported in Table 5.

**Table 5:** Pre-and post-test questionnaire data analysis.

Dimensionality	Test phase	Number of cases	Average	*p*-value
Critical thinking	Pre-test	32	1.71	0.00
	Post-test	32	2.62	
Communication	Pre-test	32	2.37	0.00
	Post-test	32	3.55	
Collaboration	Pre-test	32	2.18	0.00
	Post-test	32	3.32	
Creativity	Pre-test	32	1.56	0.00
	Post-test	32	2.43	

#### 3.2.2. Text data collection

Text data from semi-structured interviews and study notes were collected for analysis to investigate the cognitive development structure of participants during interdisciplinary thematic learning, thus clarifying the cognitive development process and the acquisition of 4C skills of participants in interdisciplinary thematic learning activities. In this study, grounded theory-based qualitative analysis was used for additional coding. The preliminary data were classified. The interview questionnaire is made up of 16 open-ended questions about students’ classroom participation, knowledge construction, and perceptions of 4C competencies. Here are some examples of questions: How do you come up with project ideas? How do you deal with problems? Will you seek assistance from other participants? Table 6 shows main contents of the interview questionnaire. This questionnaire’s overall reliability coefficient is 0.83, which is greater than 0.8, indicating that it is reliable. Furthermore, the Kaiser–Meyer–Olkin (KMO) score is 0.874, which is greater than 0.7, indicating that the questionnaire has good validity and may be utilized as a study measuring tool.

Researchers evaluated and summarized text data verbatim after data collection, providing a basic conceptual perspective of the entire content of interdisciplinary topic learning. Following repeated reading, modification, and comparison, early conceptual categories were constructed, establishing the groundwork for the construction of later generic relations (Yongqi, 2022). Specifically, the original text data was preliminarily sorted out at this point. The sorted text was then entered into NVivo software for particular coding analysis, yielding four first-level nodes and eight second-level nodes. The collected texts were openly coded. Coded reference points with comparable expressions were aggregated and summarized into secondary nodes during coding. As illustrated in Table 7, “problem-solving” and “self-reflection” were merged into the nodes of “behavioral performance skills, “resulting in eight secondary nodes.

During data coding, the crowd comparison method was applied. Various personnel collected and coded the data, which was subsequently assembled and compared. To eliminate the influence of subjectivity, any contradiction would be discussed and then corrected by the researchers. The saturation test of the coding findings was also performed utilizing the reserved data following data coding to ensure the correctness and tightness of the coding results.

Communication and collaboration were encoded the most frequently among the 4C skills development dimensions produced *via* coding, reaching 38 times. Furthermore, the original phrase examples in Table 7 provide additional insight into participants’ cognitive development and 4C skills development in interdisciplinary topic learning activities (Papadakis, 2021).

Furthermore, based on activity theory, this study divides learning activity units into four categories: activity objectives, learning forms, supporting instruments, and learning feedback. Students’ behavior activities are classified and evaluated by repeatedly viewing recorded classroom footage of students in order to determine how students’ 4C skills develop. Table 8 shows the precise coding condition.

The proportion of learning objectives at the communication level in this interdisciplinary themed learning activity is 47.88% based on the coding scenario of classroom recordings. Cooperative learning objectives were ranked second at 19.98%, followed by critical and creative goals at 15.63 and 13.17%, respectively. Students clearly communicate often during this interdisciplinary theme learning assignment. Furthermore, in terms of learning forms, student–student interaction accounts for approximately 50.23%, followed by teacher-student interaction, which accounts for approximately 38.34%, and students with individual students and teachers as the main body, which accounts for 6.3 and 5.13%, respectively. This implies that students are studied further as the main body in this interdisciplinary themed learning activity, and interpersonal linkages are strengthened. In terms of learning reflection and summary, 78.44% of students participate in reflective and summary learning activities, indicating that the majority of students can reflect and synthesis learning experiences beyond class to progress their own cognitive growth. 21.56% of students did not complete a reflective summary, indicating that students’ critical reflection in learning activities should be emphasized more.

**Table 6:** Main contents of the interview questionnaire.

**Dimensionality**	**Title numbers**	**Main contents**
Critical thinking	4	Reflect on what is taught
Communication	3	Communication frequency, the harvest of communication, etc.
Collaboration	4	Cooperation situation, cooperation mode, etc.
Creativity	5	Creative expression, whether new ideas are generated, etc.

**Table 7:** Coding situation.

**The primary node**	**The secondary node**	**Coded reference point**	**Original statement example**
Critical thinking	Knowledge of reflection	19	T1:Are all kinds of disaster weather harmful?
	Performance criticism	17	T1:During my studies, I did not try to solve the difficulties I encountered.
Communication	Exchange of subject knowledge	22	T1:Information related to disaster weather was shared with the same group.
	Exchange extensibility knowledge	16	T1:In the process of learning, I popularized the goal of sustainable development to my classmates.
Collaboration	Knowledge collaboration	25	T1:With the modeling knowledge learned before, I designed a disaster weather model for my group.
	Practice collaboration	13	T1:The designed disaster weather model was made by hand.
Creativity	Creative ideas	16	T1:Try to make a typhoon model with paper whirlpools.
	Creative practice	12	T1:During the practice, a special material was used to make the model.

**Table 8:** Learn the activity coding table.

Learning skill objective	Critical Thinking	Students are able to think critically about relevant knowledge and make suggestions.
	Communication	Students can communicate with others.
	Collaboration	Students are able to collaborate with others in practice.
	Creativity	Students can come up with innovative ideas.
Learning form	Teacher subject	The students study according to the teacher’s guidance.
	Individual student	Students’ independent research.
	Community of teachers and students	Students actively interact and explore with teachers.
	Interaction between students and students	Student to student interaction, such as discussion, etc
Support tool	Traditional tool	Students only use books, teaching materials, etc
	Mobile terminal	Students can actively use other intelligent tools to explore.
Learning feedback	Learning summary	Students can actively reflect and summarize in time.
	Unreflective summary	After learning, students fail to summarize and reflect on relevant knowledge.

## 4. Discussion

Problem-based interdisciplinary thematic learning exercises were devised in this study to assist students in developing 4C skills. In this section, we go through their potential for boosting skill development in greater depth. Students demonstrated improved ability in cognitive, affective, and behavioral dimensions, as well as social skills. This outcome is consistent with past research findings (Hasni et al., 2016; Guo et al., 2020).

### 4.1. Questioning and criticizing promotes critical thinking

We discovered that most students’ self-perceived critical thinking abilities improved after evaluating pre-and post-test data. This study is similar with the findings of Charlton and Avramides, who believed that adding real-world situations into learning activities could assist students in developing critical thinking skills (Charlton and Avramides, 2016). Students are encouraged to assess their ideas and improve their critical thinking skills by using real-world challenges (Hu et al., 2020). We presented the physical model and qualitatively described students’ critical thinking progress in this study.

We thoroughly examined the encoded text data and Classroom video to see how participants’ critical thinking was improved. We observed participant A as she considered whether all weather had negative impacts based on the environmental impact of disaster weather. When learning knowledge ideas, participant A raised this issue and opened communication and debate with other participants. Furthermore, during the catastrophe weather visual data analysis, the teacher presented inaccurate data for students to discuss. Some students analyzed the data attentively. Finally, in the fine arts course, students were forced to experiment developing modeling images into stereoscopic physical models. Despite the fact that they did not come up with solutions, they tried a variety of ways following critical thinking. For example, using his newly gained information, student C attempted to make model diagrams using several imaginative ways. In other words, he practiced using planar models and physical models based on prior knowledge.

The analysis of students’ cognitive growth process presented above demonstrated how interdisciplinary learning activities based on real-world challenges encourage their critical thinking. Interdisciplinary topic learning activities aided students’ thinking and exploration in both circumstances. When differences between data and intuition developed, students not only questioned the validity of the generated model or data, but also sought explanations to defend their results.

Difficulties are introduced into the human cognitive process through interdisciplinary thematic learning activities based on real-world problems. Students might begin with difficulties, then use their minds to produce active cognitive behaviors. From judging “knowledge” to judging their own “cognitive, “they can reach the dimension of applying talents to better their mind and spirit. Students may question and think critically, as well as accurately and purposefully correlate actions and outcomes. Students gain proficiency in spotting difficulties and asking questions in complex circumstances by gathering evidence to analyze, reason, judge, evaluate, self-adjust, and make a choice based on prior experience. Students actively explore and form conclusions using the thinking process as a means of inquiry. In a nutshell, the act of problem solving cultivates students’ critical thinking abilities. Furthermore, students gain the ability to identify and analyze problems, create judgments and formulate plans, as well as the habit of thinking about and probing into difficulties, which stimulates their critical thinking.

### 4.2. Open practice promotes the cultivation of creative thinking

We discovered that most students’ self-perceived creative thinking capacity improved after evaluating pre-and post-test data, but the change was less significant than the improvement in the other three skills. These findings emphasize the value of problem-based interdisciplinary thematic activities in encouraging student creativity (Charlton and Avramides, 2016). Fine art activities, as revealed in the study, effectively stimulate students to think creatively, whereas open questions encourage students to conceive solutions creatively and practice them through creative expression. To begin, we watched participants in the analysis when they employed creative techniques to explore diverse modeling solutions while solving difficulties in 3D modeling. Students sought to reset the parameters to make fresh models numerous times after learning some fundamental skills of 3D modeling. This phenomena implies that individuals are not just attempting to solve problems, but are also creatively broadening their thinking in order to construct more pleasing models. Second, because interdisciplinary thematic learning stresses problem solving in the actual world, we found that students came up with a wide range of creative ideas for environmental sustainability. Importantly, some of these answers were not predicted by the teaching team, demonstrating students’ ingenuity even further. Finally, students in the art department created 3D paper sculptures by exploring the knowledge and value in the catastrophe weather data, demonstrating their ingenuity.

According to the findings of the preceding investigation, students actively explored, provided solutions, and shared their opinions in interdisciplinary topic learning activities. In an interdisciplinary thematic learning environment centered on real-world situations, creative thinking develops. The openness of the design of interdisciplinary topic learning activities may promote the development of creative thinking. For example, interdisciplinary topic learning focused on real-world challenges allows students to effortlessly and effectively examine as many designs as feasible. Furthermore, interdisciplinary thematic learning environments help students develop as designers and problem solvers. This dual identity permits students to engage in not only learning tasks but also divergent thinking in order to propose alternate answers to open-ended situations. Finally, rather of simply reporting answers to questions, students are encouraged to solve problems creatively and share their opinions. These questions are strongly tied to their life experience and future life, allowing students to envision their future lives based on previous learning experiences and increasing their feeling of social responsibility.

Creative thinking is nurtured in open practice in this interdisciplinary thematic learning activity based on real-world situations, and creative result-oriented learning is promoted to break through habitual cognitive modes and integrate schemes and converge thinking. Furthermore, by emphasizing experience, design, reflection, and integration, interdisciplinary thematic learning effectively promotes learning, improves innovative design, collaboration, and action, and contributes to creative outcomes, thereby stimulating the development of learners’ creative thinking.

### 4.3. Develop communication and collaboration as a group

The study of the data before and after the test revealed that most students’ self-perceived communication and collaboration skills improved significantly. Interdisciplinary thematic problem-solving activities promote communicative scaffolding of ideas. This results supports prior research that found that integrative thematic learning activities centered on real-world situations increase both oral and writing communication skills (Gadhamshetty et al., 2016; Clark and Mahboobin, 2017). This also suggests that when students are able to share their thoughts with others, they can acquire social communication skills (Owens and Hite, 2020). Students were able to actively participate in collaborative debugging during the previous class, demonstrating that problem-solving exercises can increase students’ collaboration (Gadhamshetty et al., 2016).

Encoded text data and Classroom video were examined in depth to see how participants’ communication and collaboration skills improved. Students presented their disastrous weather dioramas in Lesson 5. Furthermore, students actively performed numerous activities in each course through communication and collaboration. Students, for example, actively participated in the collaborative debugging activity during catastrophic weather modeling in Lesson 4. When creating the stereo model, Student D discovered that the model was not solid. Then, other students debated ways to strengthen the model. They came up with a viable answer after only 2 min of deliberation.

The findings suggest that communication and teamwork skills can be developed in an interdisciplinary learning environment focused on real-world challenges. Interdisciplinary thematic learning activities need the engagement of numerous parties and the development of cooperative groups in the cross-border method of individuals and groups, emphasizing “communication optimization and action generating.” Learning activities should be carried out with the concept of “accumulating information from experience and learning from actual operation” in mind. Students should be able to dive deeper into the process of thinking, finding, and solving problems if communication is maintained throughout the learning activities. Students can improve their abilities to acquire information, communicate and collaborate, and solve problems with complete knowledge in this manner.

Students develop the learning habit of inquiry and are effective at spotting and asking questions in a complicated setting through this interdisciplinary thematic learning exercise based on real-world challenges and driven by critical thinking. Accurately expressing the problem analysis outcomes assists students in developing the habit of effective communication. Cooperation in a group encourages students to tackle challenges together and fosters the habit of collaborative work. To foster students’ inventive consciousness, spirit, and aptitude, innovative ideas and products are used as the end result of problem-solving.

## 5. Conclusion

Given society’s demand for greater interdisciplinary expertise, we should consider the following issues: What are the most significant obstacles to interdisciplinary learning? What role does interdisciplinary learning play in the development of 4C skills? As a result, the current study focused on determining whether an interdisciplinary thematic learning environment based on real-world situations might improve students’ 4C skills and problem-solving abilities.

Our study integrates real-world challenges with interdisciplinary learning in order to provide a more systematic and organized approach to teaching interdisciplinary courses. To understand the development of the 4C skills during the learning process, we conducted tests before and after the students’ self-assessment and conducted a qualitative analysis of the questionnaire answers. We found that integrative learning activities based on real-world challenges help students develop the 4C skills. Despite the limited sample size, the findings imply that interdisciplinary thematic learning centered on real-world challenges can help learners improve their 4C skills. A “common topic” across disciplines, guided by real-world challenges, can be utilized as the “common thread” to connect all discrete knowledge and skills, give a space and platform for students to employ knowledge and skills, and inspire them to mobilize knowledge and skills to think and explore. The interdisciplinary thematic learning course for growing “4C Skills” is built on transboundary knowledge as a reconstruction of the traditional curriculum system, and it serves as a model for the design of interdisciplinary thematic learning courses directed to the development of 4C skills.

The study, however, had several drawbacks. For starters, it only covers second-year students from one Chinese middle school. Regionally, respondents in this survey may have higher levels of 4C skills than students in other regions. Second, subjective factors may have an impact on this study. As a result, students’ pre-and post-test data can only be utilized as a guide. Furthermore, the pre-test is used as the baseline for reference and comparison in this work, and students are not permitted to participate in any other extracurricular activities during the study time. Other factors, however, may influence the learning process, and the development of 4C skills cannot be entirely attributed to this course. The majority of students’ self-perceived 4C skills have improved, however some students’ self-perceived 4C skills have not changed. This study did not take into account students’ individuality. In the future, 4C skills can be researched independently to better comprehend the methodologies for cultivating 4C skills. To quantify the progress in 4C skills, more research is needed. Furthermore, longitudinal studies can be used to investigate the long-term influence of interdisciplinary thematic learning focused on real-world challenges on students’ 4C skills development.

## Data availability statement

The original contributions presented in the study are included in the article/supplementary material, further inquiries can be directed to the corresponding author.

## Ethics statement

Written informed consent was obtained from the individual(s) for the publication of any potentially identifiable images or data included in this article.

## Author contributions

All authors listed have made a substantial, direct, and intellectual contribution to the work and approved it for publication.

## Conflict of interest

The authors declare that the research was conducted in the absence of any commercial or financial relationships that could be construed as a potential conflict of interest.

## Publisher’s note

All claims expressed in this article are solely those of the authors and do not necessarily represent those of their affiliated organizations, or those of the publisher, the editors and the reviewers. Any product that may be evaluated in this article, or claim that may be made by its manufacturer, is not guaranteed or endorsed by the publisher.

## References

[ref1] Ackermann Edith K. (2013). Microgenetic learning analysis: a methodology for studying knowledge in transition: commentary on Parnafes and DiSessa. Hum. Dev. 56, 38–46. doi: 10.1159/000345540

[ref2] Beane J. A. (1995). Curriculum integration and the disciplines of knowledge. Service Learning General 44, 616–622.

[ref3] Chan Z. C. Y. (2013). Exploring creativity and critical thinking in traditional and innovative problem-based learning groups. J. Clin. Nurs. 22, 2298–2307. doi: 10.1111/jocn.12186, PMID: 23452036

[ref4] Charlton P. Avramides K. (2016). Knowledge construction in computer science and engineering when learning through making. IEEE Trans. Learn. Technol. 9, 379–390. doi: 10.1109/TLT.2016.2627567

[ref5] Chen C. Lin J. (2019). A practical action research study of the impact of maker-centered STEM-PjBL on a rural middle school in Taiwan. Int. J. Sci. Math. Educ. 17, 85–108. doi: 10.1007/s10763-019-09961-8

[ref6] Clark R. M. Mahboobin A. (2017). Scaffolding to support problem-solving performance in a bioengineering lab–a case study. IEEE Trans. Educ. 61, 109–118. doi: 10.1109/TE.2017.2755601

[ref7] Czerniak C. M. Weber W. B. Sandmann J. A. Ahern J. (1999). Literature review of science and mathematics integration. Sch. Sci. Math. 99, 421–430. doi: 10.1111/j.1949-8594.1999.tb17504.x

[ref8] Dym C. L. Agogino A. M. Eris O. Frey D. D. Leifer L. J. (2005). Engineering design thinking, teaching, and learning. J. Eng. Educ. 94, 103–120. doi: 10.1002/j.2168-9830.2005.tb00832.x

[ref9] Engeström Y. (1987). Learning by Expanding: An Activity-theoretical Approach to Developmental Research (1st). Helsinki: Orienta-Konsultit.

[ref10] English L. D. King D. Smeed J. (2017). Advancing integrated STEM learning through engineering design: sixth-grade students’ design and construction of earthquake resistant buildings. J. Educ. Res. 110, 255–271. doi: 10.1080/00220671.2016.1264053

[ref11] European Council. (2018). Council recommendation of 22 May 2018 on key competences for lifelong learning [EB/OL] 2018-05-23, 2022-03-28. Available at: http://data.consilium.europa.eu/doc/document/ST-9009-2018-INIT/EN/pdf

[ref12] Ferreira M. M. Trudel A. R. (2012). The impact of problem-based learning (PBL) on student attitudes toward science, problem-solving skills, and sense of community in the classroom. J. Classr. Interact. 47, 23–30.

[ref13] Gadhamshetty V. Shrestha N. Kilduff J. E. (2016). Project-based introduction to an engineering design course incorporating microbial fuel cells as a renewable energy technology. J. Prof. Issues Eng. Educ. Pract. 142:05016001. doi: 10.1061/(ASCE)EI.1943-5541.0000272

[ref14] Gao X. Li P. Shen J. Sun H. (2020). Reviewing assessment of student learning in interdisciplinary STEM education. Int. J. STEM Educ. 7:24. doi: 10.1186/s40594-020-00225-4

[ref15] Gardner J. E. Wissick C. A. Schweder W. Canter L. S. (2003). Enhancing interdisciplinary instruction in general and special education: thematic units and technology. Remedial Spec. Educ. 24, 161–172. doi: 10.1177/07419325030240030501

[ref16] Ghefaili A. (2003). Cognitive apprenticeship, technology, and the contextualization of learning environments. J. Educ. Comput. Des. Online Learn. 4, 1–27. doi: 10.9774/GLEAF.978-1-909493-38-4_2

[ref17] Griese B. Lehmann M. Roesken-Winter B. (2015). Refining questionnaire-based assessment of stem students’ learning strategies. Int. J. STEM Educ. 2:12. doi: 10.1186/s40594-015-0025-9

[ref18] Guo P. Saab N. Post L. S. Admiraal W. (2020). A review of project-based learning in higher education: student outcomes and measures. Int. J. Educ. Res. 102:101586. doi: 10.1016/j.ijer.2020.101586

[ref19] Hasni A. Bousadra F. Belletête V. Benabdallah A. Nicole M. Dumais N. (2016). Trends in research on project-based science and technology teaching and learning at K-12 levels: a systematic review. Stud. Sci. Educ. 52, 199–231. doi: 10.1080/03057267.2016.1226573

[ref20] Helle L. Tynjälä P. Olkinuora E. (2006). Project-based learning in post-secondary education–theory, practice and rubber sling shots. High. Educ. 51, 287–314. doi: 10.1007/s10734-004-6386-5

[ref21] Herro D. Quigley C. Andrews J. Delacruz G. (2017). Co-measure: developing an assessment for student collaboration in steam activities. Int. J. STEM Educ. 4:26. doi: 10.1186/s40594-017-0094-z, PMID: 30631682 PMC6310374

[ref22] Hu C. C. Yeh H. C. Chen N. S. (2020). Enhancing STEM competence by making electronic musical pencil for non-engineering students. Comput. Educ. 15:103. doi: 10.1016/j.compedu.2020.103840

[ref23] Igira F. T. Gregory J. (2009). Cultural Historical Activity Theory. Pennsylvania: IGI Global.

[ref24] Jacobs H. H. (1989). Interdisciplinary Curriculum: Design and Implementation. Alexandria: Association for Supervision and Curriculum Development.

[ref25] Kokotsaki D. Menzies V. Wiggins A. (2016). Project-based learning: a review of the literature. Improv. Sch. 19, 267–277. doi: 10.1177/1365480216659733

[ref26] Levy S. (1992). Artificial Life: A Report from the Frontier where Computers meet Biology. New York: Random House Inc.

[ref27] Lin K. Y. Yu K. C. Hsiao H. S. Chang Y. S. Chien Y. H. (2020). Effects of web-based versus classroom-based STEM learning environments on the development of collaborative problem-solving skills in junior high school students. Int. J. Technol. Des. Educ. 30, 21–34. doi: 10.1007/s10798-018-9488-6

[ref28] Meng C. (2022). What is and what can be done in interdisciplinary thematic learning. Basic Educ. Curric. 11, 4–9. doi: 10.3969/j.issn.1672-6715.2022.11.002

[ref29] Miller E. C. Krajcik J. S. (2019). Promoting deep learning through project-based learning: a design problem. Discip. Interdis. Sci. Educ. Res. 1:7. doi: 10.1186/s43031-019-0009-6 PMC8325995 38624892

[ref30] Nakakoji Y. Wilson R. (2020). Interdisciplinary learning in mathematics and science: transfer of learning for 21st century problem solving at university. J. Intelligence 8:32. doi: 10.3390/jintelligence8030032, PMID: 32882908 PMC7555771

[ref31] Nardi B. A. (1996). Context and Consciousness: Activity Theory and Human-Computer Interaction. Cambridge: The MIT Press.

[ref32] Organization for Economic Cooperation and Development. (n.d.). The definition and selection of key competencies: executive summary [EB/OL]. (2005-05-27) [2022-03-26]. Available at: http://www.oecd.org/dataoecd/47/61/35070367.pdf

[ref33] Owens A. D. Hite R. L. (2020). Enhancing student communication competencies in STEM using virtual global collaboration project-based learning. Res. Sci. Technol. Educ. 40, 76–102. doi: 10.1080/02635143.2020.1778663

[ref34] Papadakis S. (2021). The impact of coding apps to support young children in computational thinking and computational fluency. A literature review. Front. Educ. (6, 657895. doi: 10.3389/feduc.2021.657895.

[ref35] Papadakis S. Kalogiannakis M. Gözüm A. I. C. (2022). STEM, STEAM, computational thinking, and coding: evidence-based research and practice in children's development. Front. Psychol. 13:1110476. doi: 10.3389/fpsyg.2022.1110476, PMID: 36582327 PMC9793798

[ref36] Pardamean B. (2012). Measuring change in critical thinking skills of dental students educated in a PBL curriculum. J. Dent. Educ. 76, 443–453. doi: 10.1002/j.0022-0337.2012.76.4.tb05276.x, PMID: 22473556

[ref1002] Partnership for 21st Century Learning. (2015). P21Framework Definitions. [On-line]. Available at: http://www.p21.org/storage/documents/docs/P21_Framework_Definitions_New_Logo_2015.pdf

[ref37] Partnership for 21st Century Skills. (2007). P 21 framework definitions. Available at: http://www.p21.org/about-us/p21-framework.2007-03

[ref38] Pellegrino J. W. Hilton M. L. (2012). Developing transferable knowledge and skills in the 21st century. Washington, DC: National Research Council.

[ref39] Pintrich P. Smith D. A. F. Garcia T. F. McKeachie W. J. (1991). A manual for the use of the motivated strategies for learning questionnaire (MSLQ). Washington: Office of Educational Research and Improvement.

[ref41] Richardson K. A. Cilliers P. Lissack M. (2001). Complexity science: a “gray” science for the “stuff in between”. Emergence 3, 6–18. doi: 10.1207/S15327000EM0302_02

[ref42] Roehrig G. H. Dare E. A. Ring-Whalen E. Wieselmann J. R. (2021). Understanding coherence and integration in integrated STEM curriculum. Int. J. STEM Educ. 8:2. doi: 10.1186/s40594-020-00259-8

[ref43] Sannino A. Ellis V. (2014). Learning and Collective Creativity: Activity-theoretical and Sociocultural Studies. London: Routledge.

[ref44] Shen J. Liu O. Sung S. (2014). Designing interdisciplinary assessments in sciences for college students: an example on osmosis. Int. J. Sci. Educ. 36, 1773–1793. doi: 10.1080/09500693.2013.879224

[ref45] Siegler R. S. (2007). “Handbook of child psychology” in Cognition, Perception, and Language, vol. 2. 6th ed (Hoboken, NJ: Wiley)

[ref46] Silber-Varod V. Eshet-Alkalai Y. Geri N. (2019). Tracing research trends of 21st-century learning skills. Br. J. Educ. Technol. 50, 3099–3118. doi: 10.1111/bjet.12753

[ref47] Tessier V. Zahedi M. (2022). “Activity theory as a framework for understanding framing complexity of design projects” in DRS 2022. eds. Lockton D. Lenzi S. Hekkert P. Oak A. Sádaba J. Lloyd P. (Bilbao, Spain)

[ref48] Ulger K. (2018). The effect of problem-based learning on the creative thinking and critical thinking disposition of students in visual arts education. Interdisc. J. Prob. Learn. 12. doi: 10.7771/1541-5015.1649

[ref49] Van Laar E. Van Deursen A. J. Van Dijk J. A. De Haan J. (2017). The relation between 21st-century skills and digital skills: a systematic literature review. Comput. Hum. Behav. 72, 577–588. doi: 10.1016/j.chb.2017.03.010

[ref50] Voogt J. Roblin N. P. (2012). A comparative analysis of international frameworks for 21st century competences: implications for national curriculum policies. J. Curric. Stud. 44, 299–321. doi: 10.1080/00220272.2012.668938

[ref51] Wang H. H. Charoenmuang M. Knobloch N. A. Tormoehlen R. L. (2020). Defining interdisciplinary collaboration based on high school teachers’ beliefs and practices of STEM integration using a complex designed system. Int. J. STEM Educ. 7:3. doi: 10.1186/s40594-019-0201-4

[ref52] Weng X. Cui Z. Ng O. L. Jong M. S. Y. Chiu T. K. F. (2022). Characterizing students’ 4C skills development during problem-based digital making. J. Sci. Educ. Technol. 31, 372–385. doi: 10.1007/s10956-022-09961-4

[ref53] Wenzheng Y. Qiuxuan X. (2021). STEAM interdisciplinary teaching with big concepts: model building and practice cases. J. Dist. Educ. 39, 103–112. doi: 10.15881/j.cnki.cn33-1304/g4.2021.02.011

[ref55] Xinning P. Xinyang L. (2013). Rebuilding education for the 21st century -- the establishment of the Core literacy framework of the European Union. Glob. Educ. Perspect. 42, 89–102.

[ref56] Yang H. L. Cheng H. H. (2010). Creativity of student information system projects: from the perspective of network embeddedness [J]. Comput. Educ. 1, 209–221. doi: 10.1016/j.compedu.2009.08.004

[ref1001] Yongqi X. (2022). Construction of Target System of Primary and Secondary School Labor Education in New Era -- Qualitative Analysis Based on Grounded Theory [J]. Shanghai Education Scientific Research 417, 47–51 + 37. doi: 10.16194/j.cnki.31-1059/g4.2022.02.008

[ref57] You H. S. Marshall J. A. Delgado C. (2018). Assessing students’ disciplinary and interdisciplinary understanding of carbon cycling. J. Res. Sci. Teach. 55, 377–398. doi: 10.1002/tea.21423

[ref58] Yushun L. Luqi T. Xue G. Bin S. Ying Z. (2022). Elementary school mathematics classroom teaching activity model construction based on activity theory [J]. China audio-visual. Education 427, 61–67. doi: 10.3969/j.issn.1006-9860.2022.08.008

[ref59] Zhanghua (2017). Interdisciplinary learning: true meaning and practical approach. Manag. Prim. Sec. Schools 11, 21–24.

[ref60] Zhao Huiqin W. Zhaoxue Z. T. (2019). Maker curriculum design and development for the cultivation of 4C ability in the intelligent age -- implementation path based on STEAM concept. J. Dist. Educ. 37, 104–112. doi: 10.15881/j.cnki.cn33-1304/g4.2019.01.010

[ref61] Zhao Y. Wang (2022). A case study of student development across project-based learning units in middle school chemistry. Discip. Interdscip. Sci. Educ. Res. 4:5. doi: 10.1186/s43031-021-00045-8

